# Strategies in Diagnosis and Therapy of External Outflow Graft Obstruction in Patients with a Fully Magnetically Levitated Left Ventricular Assist Device: A Meta-Analysis and Systematic Review

**DOI:** 10.3390/jcm14010108

**Published:** 2024-12-28

**Authors:** Anna Huang, Johanna K. R. von Mackensen, Vanessa I. T. Zwaans, Carla L. Schuering, Jasper Iske, Julia Stein, Sascha Ott, Roland Heck, Christoph T. Starck, Joerg Kempfert, Stephan Jacobs, Volkmar Falk, Evgenij V. Potapov, Leonhard Wert

**Affiliations:** 1 Department of Cardiothoracic and Vascular Surgery, Deutsches Herzzentrum der Charité, 13353 Berlin, Germanyleonhard.wert@dhzc-charite.de (L.W.); 2 Department of Cardiothoracic Surgery, Charité—Universitätsmedizin Berlin, Corporate Member of Freie Universität Berlin and Humboldt-Universität zu Berlin, 10117 Berlin, Germany; 3 German Center for Cardiovascular Research (DZHK), 10785 Berlin, Germany; 4 Department of Health Sciences and Technology, ETH Zürich, 8093 Zurich, Switzerland

**Keywords:** left ventricular assist device, HeartMate 3, outflow graft, external outflow graft obstruction, outflow graft compression, biodebris, outflow graft tamponade

## Abstract

**Background:** The HeartMate 3 (HM3, Abbott) left ventricular assist device (LVAD) is the only commercially available option considered suitable for long-term circulatory support. External compression of the outflow graft causing obstruction (eOGO) is a serious adverse event affecting patients on long-term support. The obstruction occurs due to the accumulation of gelatinous substance between the bend relief and outflow graft. This systematic review evaluated all available studies with regard to the diagnosis and therapy of eOGOs. **Methods:** A systematic literature review and analysis of individual patient data was performed using MEDLINE/PubMed following PRISMA guidelines. Original works dating up to 31 October 2024 were considered. **Results:** Twenty-four publications that met the inclusion criteria were identified, providing individual data from 113 patients with a median support time to eOGO diagnosis of 809 days [588, 1095] and follow-up after treatment of 365 days [33, 605]. eOGO severity classification was performed on 108 patients according to our grading system. For treatment, most patients underwent surgery (*n* = 38) or stenting (*n* = 29). A hazard ratio of 0.75 [0.28, 2.03] was calculated for the surgical group versus the stenting group (*p* = 0.570). **Conclusions:** Concerning 30-day mortality, we did not find a significant difference between the eOGO severity of survivors and non-survivors. We found no significant differences in outcome between patients with differing eOGO severity and treatment strategy, namely stenting and surgery. Due to an increase in eOGO incidence after one year of support, we propose that clinicians monitor their patients for this complication when support time surpasses one year.

## 1. Introduction

The HeartMate 3 (HM3; Abbott, Abbott Park, IL, USA) is a fully magnetically levitated centrifugal-flow left ventricular assist device (LVAD) which, in terms of hemocompatibility, is superior to both its predecessors, the axial-flow HeartMate 2 (HM2; Abbott, Abbott Park, IL, USA) and the HVAD (Medtronic, Minneapolis, MN, USA) LVAD system [1,2].

**Figure 1:**
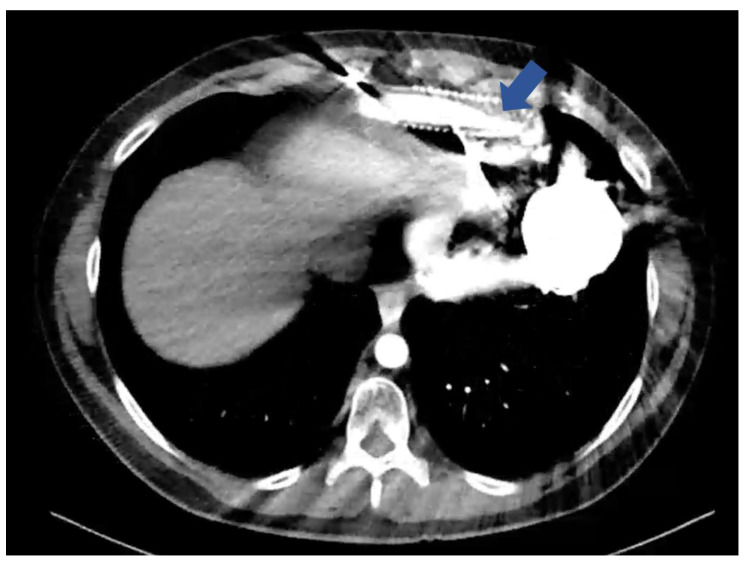
Contrast-enhanced CT-angiography of a patient with eOGO (blue arrow). CT, computed tomography; eOGO, external compression of the outflow graft causing obstruction.

Currently, the predominant complication in long-term HM3 support patients is thought to be external compression of the outflow graft causing obstruction (eOGO), with reported incidences of up to 30% [3,4]. eOGO is a potentially fatal complication that occurs due to an accumulation of gelatinous substance between the outflow graft and the bend relief compressing the outflow graft [3,5,6,7]. The porous nature of the polyethylene terephthalate (PET) outflow graft allows proteinaceous blood components to seep through [8,9]. Because the polytetrafluoroethylene (Gore-Tex) bend relief is impermeable, the proteinaceous blood components are trapped. The trapped blood components progressively accumulate in the 2 mm circumferential space between the bend relief and the outflow graft, forming a gelatinous substance. This gradual build-up of gelatinous substance eventually causes outflow graft compression and luminal narrowing, subsequently impairing the flow rate (Figure 1) [5,10,11,12]. In February 2024, Abbott released an Urgent Field Safety Notice addressing this complication, stating that design modifications for the HM3 devices will be implemented to minimize the accumulation of gelatinous substance between the outflow graft and the bend relief, possibly preventing eOGO formation in the future [13].

An accurate assessment of the impact of eOGO on long-term HM3 support patients has thus far been challenging due to a lack of standardized screening guidelines, with cases of eOGO being diagnosed coincidentally or even going undetected [5,10,14,15]. Therefore, the prevalence and incidence of eOGO might be underestimated due to the large number of undiagnosed cases.

Furthermore, there is no standard treatment strategy. Several interventional methods are available with no clear indication as to which yields a better outcome [5].

**Figure 2:**
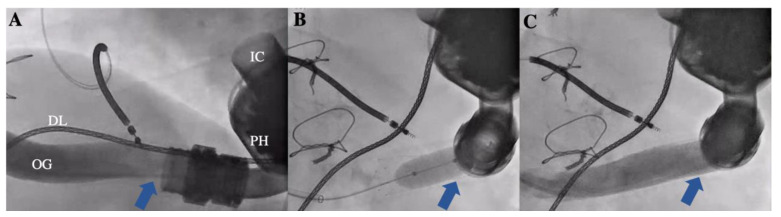
Percutaneous angiography of a patient with eOGO. (**A**) Preinterventional angiography of the compressed outflow graft due to eOGO (blue arrow). (**B**) Stent implantation and balloon dilatation (blue arrow). (**C**) Postprocedural angiography of outflow graft with improved flow (blue arrow). DL, driveline; eOGO, external compression of the outflow graft causing obstruction; IC, inflow cannula; OG, outflow graft; PH, pump housing.

Surgical methods include replacing the HM3 device and opening the bend relief to remove the gelatinous substance causing the eOGO. Percutaneous stenting to dilate the luminal diameter of the outflow graft caused by the eOGO is also frequently performed (Figure 2) [3,4,5,7,10,11,16,17,18,19,20,21,22,23,24,25].

eOGO patients who have received urgent heart transplantation have the best outcome, with no deaths reported in the published data [5,7,26,27]. However, this is not a readily accessible treatment option due to the highly limited pool of donor hearts [28].

The available literature contains systematic reviews evaluating the outcomes of eOGO patients with HM3 devices undergoing percutaneous stenting [29,30]. To our knowledge, however, the outcomes of surgically treated and stented patients have not yet been compared. To evaluate and compare different diagnostic and treatment strategies for eOGO in HM3 patients, we performed a systematic review and analysis of the existing literature.

## 2. Materials and Methods

This systematic review was conducted and reported following the Preferred Reporting Items for Systematic Reviews and Meta-Analyses (PRISMA) and has not been registered [31]. Details concerning the protocol of this systematic review and meta-analysis have not been published elsewhere. The aim of this study is to compare the outcomes of different diagnostic and treatment strategies for eOGO in the existing literature.

### 2.1. Literature Search Strategy

An electronic search was performed in October 2024 using PubMed to screen eligible studies published from 2015 onwards (when the HM3 system was first approved for both short- and long-term support). The following mesh terms were used: ‘left ventricular assist device’ or ‘LVAD’ or ‘HeartMate 3’; and ‘external outflow graft obstruction’ or ‘outflow graft compression’ or ‘extraluminal compression’ or ‘external outflow graft stenosis’ or ‘outflow graft narrowing’ or ‘outflow graft occlusion’ or ‘blood flow obstruction’. The titles of the initial results were screened, and all publications matching the inclusion criteria of confirmed eOGO in HM3 patients were imported into the reference management software EndNote 20^®^ (Clarivate Analytics, London, UK). References of the screened articles were also reviewed to identify other potentially relevant studies. A manual search was also performed to identify any articles missed by the PubMed search. Duplicates of the same case reported in separate publications were identified and removed. The remaining publications were assessed for the fulfillment of the inclusion criteria. Full texts of all relevant articles were obtained and evaluated by the first author.

### 2.2. Selection Criteria

Published case reports and case series, as well as retrospective studies among HM3 patients diagnosed with eOGO were considered. eOGO was defined as outflow graft obstruction due to extraluminal compression caused by an accumulation of gelatinous substance between the outflow graft and the bend relief [5]. Inclusion required documentation of the following criteria:

1. Use of the HM3 LVAD
2. eOGO diagnosis was confirmed through computed tomography (CT) imaging, percutaneous angiography (PA), or visualization during surgery.

Patients without a reliable eOGO diagnosis were excluded, as were patients with a thrombus inside the pump, inflow graft or outflow graft, a twisted or kinked outflow graft without eOGO, and stenosis of the distal non-covered part of the outflow graft or aortic anastomosis.

Furthermore, articles not written in English or not providing primary data were excluded. Next, the risk of bias in the included studies was assessed using the JBI Critical Appraisal Checklist for Case Series/Case Reports. The overall appraisal of the included publications served to improve the interpretation of the results synthesized in this review [32,33].

### 2.3. Data Extraction

Patient-level data was extracted by the first author and checked for accuracy by the last author. The extracted data included: authors, paper title, year of publication, size of patient cohort, patient demographics (age, sex), cardiac pathology, time on LVAD support until eOGO diagnosis, therapy target, surgical access for LVAD implantation, concomitant and further/later cardiac surgeries or interventions, clinical symptoms at admission, anticoagulation at admission, platelet inhibition, device flow rate at admission and discharge, motor power at admission and discharge, serum lactate dehydrogenase at admission and discharge, international normalized ratio (INR) at admission and discharge, echocardiography parameters, CT results, PA results, days to intervention, surgical methods to treat eOGO, type of stent used in percutaneous intervention, result of eOGO treatment (improved LVAD flow, discharge home, death, recurring eOGO), follow-up time, events of death, and patients receiving heart transplantation. Patients were censored when information was unavailable.

### 2.4. Classification of eOGO

A three-tier grading system was adopted to classify the eOGO as mild, moderate, or severe according to diagnostic findings using a similar approach to Wert et al. (2024) [5].

Where available, quantitative CT assessment was used for eOGO classification, with the degree of stenosis and corresponding category outlined below (Table 1). We considered CT imaging to be the superior diagnostic modality for eOGO due to its high spatial resolution and contrast enhancement, thus providing an accurate assessment of percentage area stenosis in the outflow graft.

The same approach was used to categorize PA or intravascular ultrasound (IVUS) findings if quantitative CT assessment was not available.

Where quantitative assessment by CT, PA, or IVUS was not available, echocardiography parameters were used to assess eOGO severity, namely, left ventricular ejection fraction (LVEF) or right ventricular ejection fraction (RVEF) (Table 2). The echocardiographic assessment was considered the lowest-ranking diagnostic tool for eOGO since it does not provide direct imaging of the outflow graft and cannot confirm the diagnosis of an eOGO by itself.

If no quantitative diagnostic assessment by CT, PA, IVUS, or echocardiography was available, qualitative CT evaluation was used for eOGO classification.

### 2.5. Statistical Analysis

Available patient-level data were extracted from eligible studies and included in the statistical analysis.

Categorical variables are presented as *n* (%) and were compared using Fisher’s exact test. Continuous variables are summarized as mean ± SD or median with interquartile range [IQR] and were compared using Student’s *t*-test or the Mann–Whitney U test. Descriptive statistical analysis was performed using the data analysis tool R, Version 4.02 (R Foundation for Statistical Computing, Vienna, Austria).

A cumulative incidence curve was plotted for all eOGO patients. The associations between treatment options (namely stenting and surgery) and survival outcomes were assessed using Kaplan–Meier analysis and Cox proportional hazards model. A two-sided *p*-value < 0.05 was considered statistically significant. The proportional hazards assumption was assessed using Schoenfeld residuals testing with no indication of non-proportionality being found.

## 3. Results

### 3.1. Literature Research

**Figure 3:**
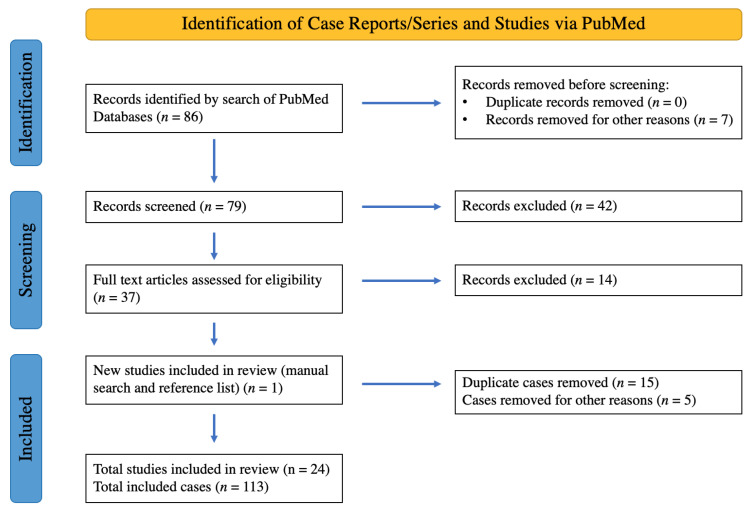
PRISMA flowchart for systematic review.

The PubMed search yielded a total of 86 publications. Of these, 37 were classified as relevant on the basis of their abstracts, and full-text reading was subsequently carried out. In the end, 24 publications were considered eligible and included in this review. Repeated reports of the same case in different publications were identified and excluded [5,7,11,22,34]. Additionally, publications reporting on multiple complications in different LVAD systems without a reliable indication of whether eOGO occurred in patients with HM3 devices were excluded [35]. Reports on patients with recurring eOGO were also excluded if they did not cover first-time diagnosis and treatment [36] (Figure 3).

**Figure 4:**
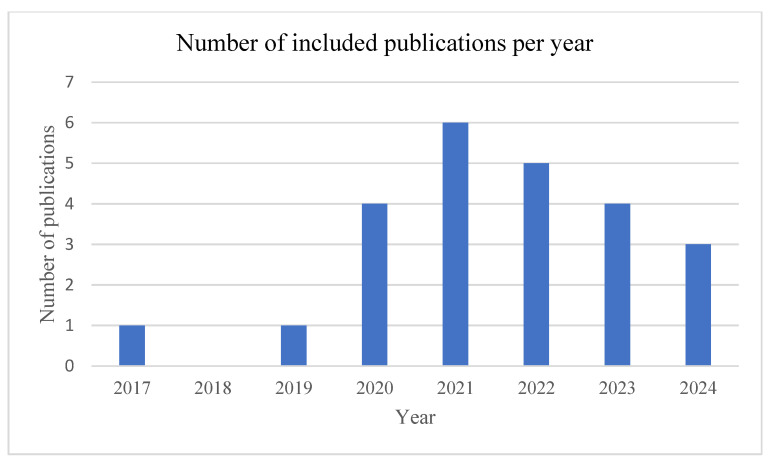
Bar chart showing the number of included publications per year on eOGO in patients on HM3 support. eOGO, external compression of the outflow graft causing obstruction; HM3, HeartMate 3.

After a full-text evaluation, publications providing data on individual cases were included, yielding a patient cohort of *n* = 113. The included studies cover a period of 8 years (Figure 4; Table S1).

The quality and quantity of information provided in the publications on the eOGO cases varied considerably. An almost complete dataset could be collected for demographics, etiology, duration of LVAD support before eOGO presentation, and clinical symptoms at eOGO manifestation. However, the collected data was also incomplete in several areas, such as surgical access used for HM3 device implantation, concomitant or further later cardiac surgeries, eOGO treatment strategy, and follow-up time (and, subsequently, the outcome of treatment).

The availability of information regarding the diagnostic parameters of eOGO patients (HM3 device flow rate, speed and motor power, serum lactate dehydrogenase (LDH), INR, and echocardiography parameters) also varied greatly.

### 3.2. Demographics and Baseline Characteristics

A dataset of 113 eOGO patients was obtained, with baseline characteristics and detailed surgical techniques used fully described in Tables S2 and S3.

Ninety-two patients (83.6%) were male. The mean age was 56 ± 13 years, and the median LVAD support duration before eOGO diagnosis was 809 days [588, 1095].

Thirty-seven patients (41.9%) suffered from ischemic and 35 patients (38.5%) from dilated cardiomyopathy. Twelve patients (13.2%) had non-ischemic cardiomyopathy without further subtype classification.

The most common therapy targets for HM3 implantation were destination therapy (*n* = 45, 54.2%) and bridge to transplant (*n* = 33, 39.8%).

Full sternotomy was performed in 58 patients (84.1%), compared to lateral thoracotomy in 11 patients (15.9%). Two patients underwent partial sternotomy together with lateral thoracotomy.

Nineteen patients had at least one concomitant surgical procedure together with the implantation of the HM3 system. In 10 of these patients, tricuspid valve reconstruction was performed concomitantly.

At least 15 patients required further cardiac surgeries at a later time. Of these, seven underwent transcatheter aortic valve replacement, four required surgical revision of the bend relief or outflow graft, and one underwent coronary artery bypass graft surgery.

### 3.3. Incidence and Clinical Presentation of eOGO

**Figure 5:**
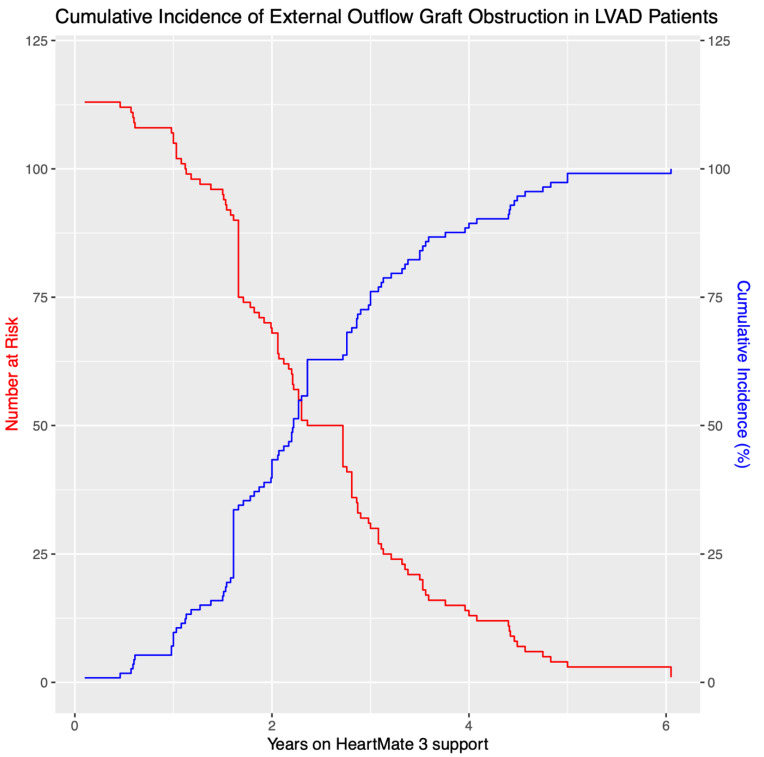
Cumulative incidence graph of eOGO obstruction in HM3 patients over 6.05 years. eOGO, external compression of the outflow graft causing obstruction; HM3, HeartMate 3.

Within the 113 eOGO patient cohort, 7.1% of patients developed eOGO within 1 year on HM3 support, 39.8% within 2 years, 74.3% within 3 years, 88.5% within 4 years, 97.3% within 5 years, and 99.1% within 6 years, with the last reported case occurring at 6.05 years (Table 3; Figure 5).

Of 107 eOGO patients with available information on clinical symptoms, 67 patients presented with device low-flow alerts (62.6%). Thirty-nine patients also presented with dyspnea as an additional or sole symptom (36.5%), while another 17 patients (15.9%) exhibited signs of heart failure or cardiogenic shock. Six patients were asymptomatic, with eOGO being diagnosed incidentally (Tables S4 and S5).

### 3.4. Diagnosis and Outcome

Ninety patients were classified by CT assessment (83.3%), five patients according to PA findings (4.6%), 11 based on echocardiography parameters (10.2%), and two according to IVUS findings. There were 22 cases (29.3%) of mild eOGO, 26 (34.7%) of moderate eOGO, and 27 (36.0%) of severe eOGO (Table S5).

Of the 27 patients with severe eOGO (48.1%), 13 subsequently underwent surgery, another nine received a stent (33.3%), two underwent transplantation, and two were managed by a watchful waiting approach (Table S6). Six patients with moderate eOGO underwent surgery, and seven received a stent. A further four patients with mild eOGO were surgically treated, and one was stented.

There was no significant difference in eOGO severity between patients undergoing stenting or surgery (*p* = 0.418).

Of 10 patients who died within 30 days after eOGO diagnosis, two had severe eOGO, and there was one case each of mild and moderate eOGO. We were unable to classify the eOGO diagnosis of the remaining six patients. We did not find significant differences in eOGO severity between 30-day survivors and non-survivors (*p* = 0.677).

### 3.5. Treatment and Outcome

The patient cohort was divided into five groups depending on the treatment they received for their eOGO: surgery, stenting, heart transplantation, watchful waiting, or intravenous (IV) anticoagulation.

Patients receiving more than one treatment (such as surgery following unsuccessful stenting or heart transplantation after previous surgery or stenting) were assigned to the group according to the last treatment option they received.

Thirty-eight patients underwent surgery, 29 patients received percutaneous stenting, 16 patients underwent heart transplantation, eight patients were assigned to the watchful waiting group, and one patient to the IV anticoagulation group (Table S6). The treatment strategy was not specified for 21 patients, who were therefore not assigned to any specific treatment group.

Due to the many cases of unspecified eOGO treatment strategy, the sample size for subsequent calculations for group comparisons decreased to *n* = 92.

Overall, we report a median follow-up time after treatment of 365 days [33, 605] and a mortality of 18.2%, with a total of 20 deceased patients. Nine patients in the surgical group (24.3%) were reported as deceased, compared to seven patients in the stenting group (25.0%).

The influence of different treatment options (namely stenting and surgery) on survival was assessed using the Cox model and Kaplan–Meier analysis. A hazard ratio (HR) of 0.75 was calculated in the surgical group versus the stenting group (confidence interval: 0.28, 2.03; *p* = 0.570).

**Figure 6:**
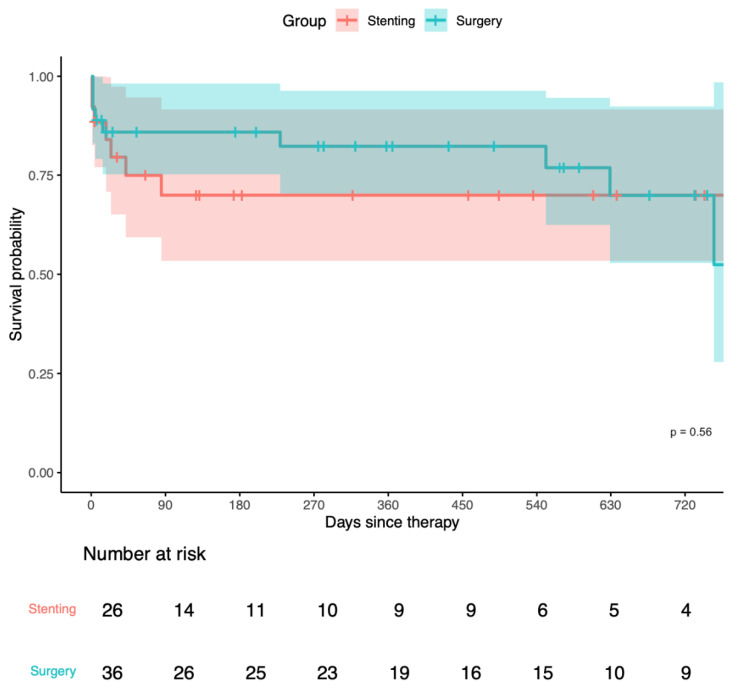
Kaplan–Meier survival curves displaying the survival probability of eOGO patients receiving different treatment options: stenting (in red) and surgery (in blue) with 95% CI indicated by shading. Follow-up time in days; *p*-value calculated by log-rank test = 0.560. eOGO, external compression of the outflow graft causing obstruction.

Furthermore, we report a 30-day mortality of 21.1% for the stenting group and 15.6% for the surgical group (*p* = 0.822). Additionally, we calculated a 90-day mortality of 33.3% for the stenting group and 16.1% for the surgical group (*p* = 0.267). The Kaplan–Meier curve showed no significant difference in survival probability between stented and surgically treated patients (*p* = 0.560) (Figure 6).

Information relating to the cause of death was available for two cases in the surgical group: Case 5 died 2 days after surgery due to multiple organ dysfunction syndrome and COVID-19 pneumonia. Case 82 died of an ischemic stroke 2 days after re-thoracotomy. Within the stenting group, Case 11 died due to cerebral hemorrhage, Case 69 due to septic shock 18 days after stenting, and Case 98 due to liver and end-organ failure 24 days after the intervention (Table S6). Details of the cause of death were not provided for other patients.

When taking into account parameters other than mortality that are indicative of treatment outcome, we found non-significant differences in LVAD flow and motor power at discharge.

Stented patients had a lower median LVAD flow at discharge of 4.50 L/min [4.30, 4.80], compared to 4.80 L/min in surgically treated patients [4.30, 5.15] (*p* = 0.234). Before treatment, median LVAD flow was 3.30 L/min [2.28, 4.10] in subsequently stented patients and 3.00 L/min [2.40, 4.00] in surgically treated patients (*p* = 0.991).

Median motor power increased to 4.15 W [3.77, 4.30] in stented patients and to 4.35 W in surgically treated patients [4.05, 4.77] after therapy (*p* = 0.074), with a median motor power at admission of 4.00 W [3.60, 4.30] and 3.85 W [3.68, 4.40], respectively (*p* = 0.698) (Tables S7 and S8).

To conclude, our data analysis does not indicate a significant difference between the outcomes in terms of mortality or LVAD parameters of patients undergoing stenting or surgical therapy for eOGO.

## 4. Discussion

Findings relating to the marked increase in eOGO incidence in HM3 patients after 1 year on LVAD support and most frequent symptoms are consistent with those reported in previous studies.

A multicenter study conducted in 2022 by Wert et al. evaluated 2108 HM3-implanted patients across 17 cardiac centers. An overall eOGO prevalence of 3.0% with a rising incidence after 1 year on HM3 circulatory support was reported [5]. Our analysis found an increase in cumulative incidence from 7.1% to 39.8% between 1 year and 2 years on HM3 support.

In terms of diagnostic assessment and outcome, we did not find significant differences in eOGO severity between 30-day survivors and non-survivors. There was also no significant difference in eOGO severity between patients receiving different treatment options (surgery or stenting).

Regarding treatment strategy and outcome, we found that surgically treated patients showed a greater but non-significant improvement than stented patients in terms of median LVAD flow and motor power: median LVAD flow increased from 3.00 [2.40, 4.00] to 4.80 L/min [4.30, 5.15] in patients undergoing surgery. Stented patients showed an increase in median flow from 3.30 [2.28, 4.10] to 4.50 L/min [4.30, 4.80]. Median motor power increased from 3.85 [3.68, 4.40] to 4.35 W [4.05, 4.77] in surgical patients. In stented patients, median motor power increased from 4.00 [3.60, 4.30] to 4.15 W [3.77, 4.30].

The available data are also insufficient to make a statement on the superiority of one treatment strategy with regard to mortality. There were no significant results regarding the differences in outcomes between patients undergoing surgery or stenting for eOGO. We suspect that this is due to the small subgroup sizes, with a further 21 patients excluded from subsequent calculations because their treatment strategy was not specified (Table S6). We would also like to note that some patients who received a heart transplantation had previously undergone stenting or surgery: Case 111 was stented, and Cases 24–26 were surgically treated and survived until they received a heart transplant (Table S6). These cases were not included in the analysis of outcome differences between patients undergoing stenting or surgery.

Another complicating factor here is that the follow-up time of the underlying case reports/series and retrospective studies varied considerably. Notably, the median follow-up time of surgically treated patients was 365 days [47.75, 690.75], more than double the median follow-up time of 129 days [19.50, 525.50] for stented patients. In some cases, it was only stated that the patient recovered and was discharged home, with no information on the patient’s condition over a longer follow-up period (Cases 28, 35, 70, 86–89, 102; Table S6). The large difference in follow-up time between surgically treated and stented patients is a relevant issue to consider regarding the 90-day mortality results: The shorter follow-up time in stented patients may result in early deaths being disproportionately emphasized, thus not accurately reflecting the long-term trend.

Abbott is currently working on a design solution to minimize the accumulation of gelatinous substance between the outflow graft and the bend relief [13]. Some implantation centers perform perforations on bend reliefs of the HM3 device prior to implantation to prevent eOGO development [34]. Other companies, such as Corvion, are also incorporating fenestrated bend relief designs into their new LVAD inventions [37]. We suspect that this will only slow down eOGO development and will not provide a permanent solution to the problem.

## 5. Limitations

We would like to highlight several factors that limit the results of this systematic review. One main factor limiting the certainty of evidence is the heterogeneous database: This systematic review reviews the largest population of eOGO patients implanted with HM3 systems to date. The basis of this review is formed predominantly by case reports or series, which introduces a high risk of reporting and selection bias. The case studies and case series included in this systematic review provide very detailed information on specific cases but do not always present variables in the same manner. There are no standardized criteria for measuring certain variables, such as eOGO severity or treatment outcome. Many publications provided a quantitative CT assessment for the degree of stenosis. However, where this was not available, we had to resort to assessment by PA, IVUS, or echocardiography to classify the eOGO. Consequently, the differences in diagnostic assessment resulted in a discrepancy in eOGO classification. We believe that using CT assessment and the three-tier grading system, as outlined in Table 1, could help reduce the discrepancy of eOGO classifications in future studies.

Publications also used different parameters as indicative of a positive treatment outcome, such as LVAD flow improvement, resolution of symptoms, or survival over a follow-up period. Several publications also failed to provide information on the treatment outcome (Case 79–80, 104; Table S6).

Finally, we acknowledge that this systematic review is limited by the scarce literature on eOGOs in HM3 patients, which might have been further exacerbated by the exclusion of non-English studies. The exclusion of non-English studies could have also introduced bias by overlooking relevant data on regional differences in eOGO incidence and management, limiting the comprehensive nature of this systematic review.

We believe that as clinicians become more aware of eOGO as a complication in long-term LVAD patients, eOGO may be reported more frequently. Therefore, it might be possible to increase the treatment subgroup sizes in future studies in order to draw more valuable conclusions about the outcomes of eOGO patients receiving differing treatments.

## 6. Conclusions

eOGO is a potentially life-threatening complication occurring in HM3 LVAD patients on prolonged support caused by an accumulation of gelatinous substance between the bend relief and the outflow graft. This systematic review analyzed the individual data of 113 patients diagnosed with eOGO. eOGO is diagnosed mostly by CT assessment, with percutaneous stenting and surgery being the most frequently used treatment options.

Due to the non-significant results in this review, we cannot draw a clear conclusion as to which treatment option is superior in terms of outcome. We would like to note the importance of individualized treatment plans, which take into consideration patient-specific factors, the experience of the LVAD implantation center, and the attending physician. However, we believe that percutaneous stenting will dominate as the preferred treatment strategy for eOGO. The general consensus is that percutaneous stenting yields a more favorable outcome than surgical methods because of its less invasive nature. Future studies featuring a larger patient cohort are needed to find sufficient evidence to support this claim. Reporting diagnostic parameters in a more standardized matter, for example, using the CT assessment grading system outlined in our study (Table 1), might help provide a more homogenous database for future studies evaluating eOGO.

## Figures and Tables

**Table 1:** Degree of stenosis evaluated using CT, PA, or IVUS and corresponding eOGO severity. CT, computed tomography; PA, percutaneous angiography; IVUS, intravascular ultrasound; eOGO, external compression of the outflow graft causing obstruction.

Degree of Stenosis	eOGO Classification
≤49%	Mild
50–74%	Moderate
≥75%	Severe

**Table 2:** Echocardiography parameters and corresponding eOGO severity. LVEF, left ventricular ejection fraction; RVEF, right ventricular ejection fraction; eOGO, external compression of the outflow graft causing obstruction.

LVEF	RVEF	eOGO Classification
≥50%	25–31%	Mild
40–49%	18–24%	Moderate
<40%	≤17%	Severe

**Table 3:** Incidence table of patients on HM3 support developing eOGO. HM3, HeartMate 3; eOGO, external compression of the outflow graft causing obstruction.

Years on LVAD Support	Incidence	Numbers at Risk	Cumulative Incidence %
1	8	113	7.1
2	37	105	39.8
3	39	68	74.3
4	16	29	88.5
5	10	13	97.3
6	2	3	99.1
7	1	1	100.0

## Data Availability

All data are publicly available. For resources, see Supplementary Material Table S1.

## Supplementary Materials

The following supporting information can be downloaded at: https://www.mdpi.com/article/10.3390/jcm14010108/s1, Table S1: List of publications reporting on eOGO as a complication in HM3 patients included in this review. Some publications report more cases than were included in the review, because they were repeated cases. eOGO, external compression of the outflow graft causing obstruction; HM3, HeartMate 3; Table S2: Demographic and baseline characteristics of published HM3 patients with eOGO (2017-2024). HM3, HeartMate 3; ASD, atrial septal defect; PFO, patent foramen ovale; RVAD, right ventricular assist device; TAVR, transcatheter aortic valve replacement; CABG, coronary artery bypass grafting; eOGO, external compression of the outflow graft causing obstruction; Table S3: Baseline characteristics of individual patients. HM3, HeartMate 3; CMP, cardiomyopathy; ICMP, ischemic cardiomyopathy, DCMP, dilated cardiomyopathy; NICMP, non-ischemic cardiomyopathy; ASD, atrial septal defect; PFO, patent foramen ovale; RVAD, right ventricular assist device; TAVR, transcatheter aortic valve replacement; CABG, coronary artery bypass grafting; eOGO, external compression of the outflow graft causing obstruction; Table S4: Symptoms displayed by eOGO patients. Information on symptom constellation was available for 107 patients (94.7% of the total patient cohort). eOGO, external compression of the outflow graft causing obstruction; Table S5: Clinical symptoms, diagnostic assessment and classification of eOGO, and laboratory parameters in individual patients. CT, computed tomography; eOGO, external compression of the outflow graft causing obstruction; Echo, echocardiography; PA, percutaneous angiography; IVUS, intravascular ultrasound; Table S6: eOGO treatment strategy, follow-up time in days, and outcome of treatment in individual patients included in this review. eOGO, external compression of the outflow graft causing obstruction; Table S7: Laboratory and LVAD parameters of eOGO patients. INR, international normalized ratio; LVAD, left ventricular assist device; eOGO, external compression of the outflow graft causing obstruction; Table S8: LVAD parameters of eOGO patients divided into therapy subgroups stenting and surgery. LVAD, left ventricular assist device; eOGO, external compression of the outflow graft causing obstruction; Video S1: Computed tomography angiography of external outflow graft obstruction; Video S2: Preinterventional percutaneous angiography of the outflow graft; Video S3: Stent placement and balloon dilatation; Video S4: Postprocedural percutaneous of the outflow graft.

## Abbreviations

ARDS	acute respiratory distress syndrome
ASD	atrial septal defect
CABG	coronary artery bypass graft
CMP	cardiomyopathy
CT	computed tomography
DCMP	dilated cardiomyopathy
eOGO	external compression of the outflow graft causing obstruction
HM3	HeartMate 3
ICMP	ischemic cardiomyopathy
INR	international normalized ratio
IVUS	intravascular ultrasound
LDH	lactate dehydrogenase
LVAD	left ventricular assist device
LVEF	left ventricular ejection fraction
NICMP	non-ischemic cardiomyopathy
PA	percutaneous angiography
PET	polyethylene terephthalate
PFO	patent foramen ovale
RVAD	right ventricular assist device
RVEF	right ventricular ejection fraction
TAVR	transcatheter aortic valve replacement
