# Supplementary material for: Strategies in Diagnosis and Therapy of External Outflow Graft Obstruction in Patients with a Fully Magnetically Levitated Left Ventricular Assist Device: A Meta-Analysis and Systematic Review
Source: J Clin Med. 2024 Dec 28;14(1):108. doi: 10.3390/jcm14010108 (PMC11720867; doi:10.3390/jcm14010108)
Supplement: Supplementary file 1 [file jcm-14-00108-s001.zip › jcm-3350977-supplementary.pdf]

Authors	*Study design*	*Journal*	*Publication year*	*Title*	*Number of cases*	*Case ID*
Akanksha Agrawal et al.	Retrospective Study	ESC Heart Failure	2021	Outflow graft obstruction after left ventricular assist device implantation: a retrospective, single-centre case series	3	86–88
Alexandra Gasecka et al.	Case Reports	EuroIntervention	2022	Percutaneous management of left ventricular assist device outflow graft obstruction	3	90–92
Andrea Montalto et al.	Case Report	Annals of Thoracic Surgery	2021	Resolution of sudden left ventricular assist device failure due to outflow graft narrowing	1	102
Assi Milwidski et al.	Retrospective Study	Catheterization and Cardiovascular Interventions	2021	Outflow graft obstruction in patients with the HM 3 LVAD: A percutaneous approach	5	66–70
Aurelie Merlo et al.	Retrospective Case Series	Journal of Thrombosis and Thrombolysis	2023	Mechanical pump complication after HeartMate 3 implantation	4	76–79
Corrado Tramontin et al.	Case Series	Asian Cardiovascular & Thoracic Annals	2022	Diagnostic and surgical management of HeartMate 3 outflow graft obstruction due to two different mechanisms	1	71
Cory R Trankle et al.	Retrospective Study	Journal of Cardiac Failure	2019	Left Ventricular Assist Device Outflow Graft Compression: Incidence, Clinical Associations and Potential Etiologies	1	80
Dor Lotan et al.	Case Report	JTCVS Techniques	2022	Recurrent outflow graft compression of HeartMate3: When the left ventricular assist device sticks to the rib	1	103
Estefania Oliveros et al.	Case Series	Journal of Cardiothoracic and Vascular Anesthesia	2024	Outflow Graft Tamponade: An Underrecognized Cause of Obstruction	8	106–113
Gaik Nersesian	Case Report	Journal of Cardiac Surgery	2020	Surgical treatment of outflow graft kinking complicated by external obstruction	1	101

				with a fibrin mass in a patient with LVAD		
Gloria Färber et al.	Retrospective Study	Interactive Cardiovascular And Thoracic Surgery	2022	Bend relief fenestration might prevent outflow graft obstruction in patients with left ventricular assist device	3	73–75
Isaac Wamala et al.	Case Reports/Imaging Vignette	JACC: Cardiovascular Imaging	2020	Computed Tomography and Fluoroscopic Angiography in Management of Left Ventricular Assist Device Outflow Graft Obstruction	1	93
Ismael A Salas De Armas et al.	Case Series	ASAIO Journal	2021	Use of a Minimally Invasive Approach to Correct Diffuse External Compression of the Left Ventricular Assist Device Outflow Graft	1	104
Jessica Rossi et al.	Case Poster	Journal of the American College of Cardiology	2024	LVAD Outflow Graft Obstruction: Rare but treatable complication	1	105
Juan G. Duero Posada et al.	Case Series	Circulation: Heart Failure	2017	Outflow Graft Occlusion of the HeartMate 3 Left Ventricular Assist Device	2	64–65
Julien Guihaire et al.	Case Report	Journal of Artificial Organs	2020	Proximal outflow graft extrinsic compression of the Heartmate 3 centrifugal flow left ventricular assist device resulting in pump dysfunction	1	81
Kamen Dimitrov et al.	Retrospective Study	European Journal of Cardio-Thoracic Surgery	2022	Incidence, clinical relevance and therapeutic options for outflow graft stenosis in patients with left ventricular assist devices	17	82–85
Leonhard Wert et al.	Multicenter Retrospective Study	Journal of Thoracic and Cardiovascular Surgery	2024	A multicenter evaluation of external outflow graft obstruction with a fully magnetically levitated left	62	1–47

				ventricular assist device		
Lu Wang et al.	Case Report	Multimedia Manual of Cardio-Thoracic Surgery	2023	Surgical intervention for left ventricular assist device outflow graft obstruction due to external compression	1	89
Paulino Alvarez et al.	Case Report	Asian Cardiovascular & Thoracic Annals	2021	Outflow graft foreign body reaction and thrombosis in HeartMate 3 left ventricular assist device	1	72
Silvia Ajello et al.	Case Series	Artificial Organs	2023	Extrinsic outflow graft flow obstruction in patients with HeartMate3 LVAD	5	94–98
Sneha S. Jain et al.	Retrospective Study	Annals of Thoracic Surgery	2023	Outflow Graft Narrowing of the HeartMate 3 Left Ventricular Assist Device	15	49–63
Yuji Kaku et al.	Retrospective Review	Interactive Cardiovascular And Thoracic Surgery	2020	Late inflow or outflow obstruction requiring surgical intervention after HeartMate 3 left ventricular assist device insertion	1	48
Zachary M Gertz et al.	Case Series	Catheterization and Cardiovascular Interventions	2021	An interventional approach to left ventricular assist device outflow graft obstruction	2	99–100

Table S1: List of publications reporting on eOGO as a complication in HM3 patients included in this review. Some publications report more cases than were included in the review, because they were repeated cases. eOGO, external compression of the outflow graft causing obstruction; HM3, HeartMate 3.

Variable	Value	Patients with available data, *n* (%)
Male, *n* (%)	92 (83.6)	110 (97.3)
Age at HM3 implantation, mean ± SD	56 (13.0)	113 (100.0)
Days on HM3 support, median [IQR]	809 [588, 1095]	97 (85.8)
Cardiac pathology, *n* (%)		91 (80.5)
Ischemic cardiomyopathy	37 (41.9)	
Dilated cardiomyopathy	35 (38.5)	
Non-ischemic cardiomyopathy	12 (13.2)	
Valvular cardiomyopathy	2 (2.2)	
Combined	2 (2.2)	
Chemotherapy-induced cardiomyopathy	1 (1.1)	
Idiopathic cardiomyopathy	1 (1.1)	
Therapy target, *n* (%)		83 (73.5)
Destination	45 (54.2)	
Bridge to transplant	33 (39.8)	
Bridge to candidacy	4 (4.8)	
Bridge to decision	1 (1.2)	
Surgical access, *n* (%)		69 (61.1)
Full sternotomy	58 (84.1)	
Lateral thoracotomy	11 (15.9)	
Partial sternotomy	2 (2.9)	
Concomitant cardiac operations, *n* (%)	19 (31.1)	61 (54.0)
Tricuspid valve reconstruction	10 (16.4)	
ASD/PFO closure	8 (13.1)	
RVAD implantation	5 (8.2)	
Aortic valve/prosthesis replacement	3 (4.9)	
Other	1 (1.6)	
Later cardiac surgeries, *n* (%)	15 (26.3)	57 (50)
TAVR	7 (12.2)	
RVAD implantation	3 (5.3)	
RVAD explantation	2 (3.5)	
CABG	1 (1.8)	
Other	5 (7.9)	
Anticoagulation, *n* (%)		66 (58.4)
Warfarin	65 (98.5)	
Fluindione	1 (1.5)	
Platelet inhibition, *n* (%)		60 (53.1)
Aspirin	42 (70.0)	
Clopidogrel	3 (5.0)	
None	15 (25.0)	

Table S2: Demographic and baseline characteristics of published HM3 patients with eOGO (2017-2024). HM3, HeartMate 3; ASD, atrial septal defect; PFO, patent foramen ovale; RVAD, right ventricular assist device; TAVR, transcatheter aortic valve replacement; CABG, coronary artery bypass grafting; eOGO, external compression of the outflow graft causing obstruction.

Case	Sex	Age at HM3 implantation	Days on support	Cardiac pathology	Therapy target	Surgical access	Concomitant cardiac surgeries	Later cardiac surgeries
1	F	64	648	Valvular CMP	Destination	Lateral thoracotomy	None	TAVR
2	M	55	752	ICMP	Bridge to transplant	Lateral thoracotomy	None	None
3	M	34	1008	DCMP	Bridge to transplant	Full sternotomy	None	None
4	M	64	1222	DCMP	Destination	Full sternotomy	None	Revision, bend relief
5	M	58	1629	ICMP	Bridge to transplant	Lateral thoracotomy	None	TAVR
6	M	46	1606	ICMP	Bridge to transplant	Lateral thoracotomy	None	None
7	M	65	1612	ICMP	Destination	Full sternotomy	None	None
8	M	61	1734	ICMP	Destination	Lateral thoracotomy	Temporary RVAD	None
9	M	58	1763	ICMP	Destination	Full sternotomy	PFO/ASD closure	Transposition of driveline, reposition of greater omentum
10	M	59	1289	ICMP	Destination	Lateral thoracotomy	None	TAVR
11	M	70	575	ICMP	Destination	Full sternotomy	None	None
12	M	59	775	Combined ICMP/DCMP	Bridge to transplant	Full sternotomy	None	None
13	M	71	792	Valvular CMP	Destination	Full sternotomy	Aortic valve prosthesis replacement	None
14	M	73	563	DCMP	Bridge to transplant	Full sternotomy	None	None
15	M	68	1234	NICMP	Destination	Full sternotomy	None	None
16	M	66	221	DCMP	Destination	Full sternotomy	None	None
17	M	13	375	Chemotherapy-induced cardiomyopathy	Bridge to transplant	Full sternotomy	Tricuspid valve reconstruction, temporary RVAD	RVAD explantation
18	M	66	806	DCMP	Destination	Full sternotomy	None	None
19	F	69	1211	DCMP	Destination	Full sternotomy	Tricuspid valve reconstruction, ASD closure	None

20	M	55	700	DCMP	Bridge to transplant	Lateral thoracotomy	None	None
21	M	52	559	ICMP	Bridge to transplant	Full sternotomy	Aortic valve replacement, tricuspid valve reconstruction	None
22	M	64	1668	ICMP	Destination	Full sternotomy	Tricuspid valve reconstruction	CABG
23	M	60	810	DCMP	Bridge to transplant	Full sternotomy	Tricuspid valve reconstruction	None
24	M	35	1311	DCMP	Bridge to transplant	Full sternotomy	Tricuspid valve reconstruction	None
25	M	50	1278	ICMP	Bridge to transplant	Full sternotomy	Left ventricular reduction	None
26	M	24	432	DCMP	Bridge to transplant	Full sternotomy	Tricuspid valve reconstruction, PFO closure	None
27	M	56	2208	DCMP	Bridge to transplant	Full sternotomy	None	None
28	M	54	665	DCMP	Destination	Full sternotomy	None	None
29	F	60	1638	DCMP	Destination	Full sternotomy	None	None
30	M	59	1173	ICMP	Destination	Full sternotomy	None	Temporary RVAD, TAVR
31	M	58	504	ICMP	Bridge to decision	Full sternotomy	None	None
32	M	55	356	DCMP	Bridge to transplant	Full sternotomy	None	None
33	M	38	1446	DCMP	Bridge to transplant	Full sternotomy	None	None
34	M	50	1059	ICMP	Bridge to transplant	Full sternotomy	None	None
35	M	58	809	DCMP	Destination	Full sternotomy	None	None
36	F	61	356	ICMP	Destination	Full sternotomy	None	None
37	F	51	624	ICMP	Destination	Full sternotomy	None	None
38	F	51	1044	ICMP	Destination	Full sternotomy	None	None
39	M	63	1299	ICMP	Destination	Partial sternotomy with lateral thoracotomy	None	None
40	F	12	407	DCMP	Bridge to transplant	Full sternotomy	None	None

41	M	38	1490	DCMP	Bridge to transplant	Full sternotomy	None	None
42	M	40	38	DCMP	Bridge to transplant	Full sternotomy	Temporary RVAD	RVAD explantation
43	M	53	1143	ICMP	Bridge to candidacy	Full sternotomy	None	Revision, outflow graft
44	M	53	804	ICMP	Bridge to transplant	Full sternotomy	Tricuspid valve reconstruction, temporary RVAD, PFO closure	RVAD explantation
45	M	71	1372	NCMP	Destination	Full sternotomy	None	None
46	M	33	841	NCMP	Bridge to transplant	Full sternotomy	None	Revision, cardiac tamponade, open chest
47	M	29	167	NCMP	Bridge to transplant	Full sternotomy	Tricuspid valve reconstruction	Revision, cardiac tamponade and temporary RVAD, open chest
48	M	42	804	DCMP	-	Full sternotomy	-	-
49	M	60.4	588	ICMP	-	-	-	-
50	M	60.4	588	ICMP	-	-	-	-
51	M	60.4	588	ICMP	-	-	-	-
52	M	60.4	588	ICMP	-	-	-	-
53	M	60.4	588	-	-	-	-	-
54	M	60.4	588	-	-	-	-	-
55	M	60.4	588	-	-	-	-	-
56	M	60.4	588	-	-	-	-	-
57	M	60.4	588	-	-	-	-	-
58	M	60.4	588	-	-	-	-	-
59	M	60.4	588	-	-	-	-	-
60	M	60.4	588	-	-	-	-	-
61	F	60.4	588	-	-	-	-	-
62	F	60.4	588	-	-	-	-	-
63	F	60.4	588	-	-	-	-	-
64	M	65	365	ICMP	Bridge to transplant	-	-	-
65	M	41	216	DCMP	Bridge to candidacy	-	-	-

66	M	72	727	Combined ICMP/valvular CMP	Destination	Full sternotomy	None	-
67	F	53	396	DCMP	-	Full sternotomy	-	-
68	F	53	365	ICMP	-	-	-	-
69	M	64	1094	NICMP	Destination	Full sternotomy	None	-
70	M	49	1608	NCMP	Bridge to transplant	Full sternotomy	None	-
71	M	72	1095	DCMP	Destination	-	-	-
72	M	65	549	ICMP	Destination	-	-	-
73	M	61	462	DCMP	Bridge to transplant	Full sternotomy	-	-
74	M	71	1043	DCMP	Destination	Full sternotomy	-	-
75	M	58	412	DCMP	Bridge to transplant	Full sternotomy	-	-
76	M	59	828	NICMP	Destination	-	-	-
77	M	59	828	NICMP	Destination	-	-	-
78	M	59	828	NICMP	Destination	-	-	-
79	M	59	828	ICMP	Destination	-	-	-
80	M	64	994	ICMP	-	-	-	-
81	M	42	219	ICMP	Bridge to transplant	Full sternotomy	-	-
82	M	70	209	DCMP	Destination	Full sternotomy	Aortic valve replacement, PFO closure	None
83	M	58	683	DCMP	Bridge to transplant	Full sternotomy	None	None
84	M	42	1124	ICMP	Bridge to candidacy	Full sternotomy	Temporary RVAD	None
85	M	71	552	ICMP	Destination	Partial sternotomy with lateral thoracotomy	None	None
86	-	46	1006	-	-	-	-	-
87	-	46	1006	-	-	-	-	-
88	-	46	1006	-	-	-	-	-

89	M	63	1278	DCMP	Bridge to candidacy	Full sternotomy	-	-
90	M	51	1460	DCMP	-	-	-	-
91	M	49	730	ICMP	-	-	-	-
92	M	54	365	ICMP	-	-	-	-
93	F	68	1049	DCMP	Destination	Lateral thoracotomy	None	None
94	M	69	607	ICMP	Destination	Full sternotomy	None	None
95	M	73	1112	ICMP	Destination	Full sternotomy	ASD closure	TAVR
96	M	74	1250	ICMP	Destination	Full sternotomy	Tricuspid valve reconstruction, ASD closure	None
97	M	78	930	DCMP	Destination	Full sternotomy	ASD closure	None
98	M	64	757	DCMP	Destination	Full sternotomy	None	TAVR
99	M	25	1095	NICMP	-	-	-	-
100	F	71	1825	NICMP	-	-	-	-
101	M	51	1825	DCMP	-	Lateral thoracotomy	-	-
102	M	49	730	Idiopathic	-	-	-	-
103	M	77	730	DCMP	Destination	Full sternotomy	-	-
104	M	27	1007	NICMP	Destination	-	None	-
105	M	59	730	ICMP	-	-	-	-
106	F	70	862	-	Destination	-	-	-
107	F	29	862	-	Bridge to transplant	-	-	-

108	F	54	862	-	Destination	-	-	-
109	M	60	862	-	Bridge to transplant	-	-	-
110	F	63	862	-	Bridge to transplant	-	-	-
111	M	36	862	-	Destination	-	-	-
112	M	44	862	-	Bridge to transplant	-	-	-
113	M	71	862	-	Destination	-	-	-

Table S3: Baseline characteristics of individual patients. HM3, HeartMate 3; CMP, cardiomyopathy; ICMP, ischemic cardiomyopathy, DCMP, dilated cardiomyopathy; NICMP, non-ischemic cardiomyopathy; ASD, atrial septal defect; PFO, patent foramen ovale; RVAD, right ventricular assist device; TAVR, transcatheter aortic valve replacement; CABG, coronary artery bypass grafting; eOGO, external compression of the outflow graft causing obstruction.

Symptom	*n* (%)
Heart failure symptoms	
Low-flow alert	67 (62.6)
Dyspnea	39 (36.5)
Cardiac decompensation	5 (5.6)
Cardiogenic shock	3 (2.8)
Hydropic decompensation	4 (3.7)
Pulmonary edema/congestion	4 (3.7)
Angina pectoris	3 (2.8)
Dizziness	1 (0.9)
Arrhythmia	1 (0.9)
Syncope	1 (0.9)
Peripheral edema	1 (0.9)
Cardiac arrest	1 (0.9)
Unspecific symptoms	
Fever/infection	2 (1.9)
Reduced vigilance	1 (0.9)
Anemia	1 (0.9)
Hyperglycemia	1 (0.9)
Renal failure	1 (0.9)
Acute respiratory syndrome	1 (0.9)
Dyspepsia	1 (0.9)
Epistaxis	1 (0.9)
Asymptomatic	6 (5.6)

Table S4: Symptoms displayed by eOGO patients. Information on symptom constellation was available for 107 patients (94.7% of the total patient cohort). eOGO, external compression of the outflow graft causing obstruction.

Case	Clinical symptoms at admission	LDH at admission	INR at admission	Diagnostic assessment	eOGO classification
1	Low-flow alert	201	2.2	CT	Moderate
2	Dyspnea, angina pectoris	195	2.5	PA	Severe
3	Incidental finding (transplant re-evaluation)	298	2.5	Echo	Severe
4	Low-flow alert, anemia	217	2	CT	Moderate
5	Low-flow alert	500	2.05	CT	Severe
6	Low-flow alert	297	3.3	Echo	Severe
7	Low-flow alert	329	1.1	Echo	Severe
8	Reduced vigilance, hyperglycemia	159	1.1	CT	Moderate
9	Hydropic decompensation	228	2.6	PA	Severe
10	Low-flow alert, dyspnea, hydropic decompensation	214	2.8	Echo	Severe
11	Low-flow alert	216	2.4	CT	Moderate
12	Dyspnea	85	2.8	CT	Moderate
13	Dyspnea	305	2.3	Echo	Severe
14	Low-flow alert	276	2.1	Echo	Severe
15	Dyspnea, low-flow alert	167	2.7	Echo	Mild
16	ARDS	330	2.82	CT	-
17	Low-flow alert	254	2.28	Echo	Moderate
18	None	-	2.31	CT	
19	Dyspnea, cardiac arrhythmia	227	1.56	CT	-
20	Low-flow alert	384	2	CT	-
21	Low-flow alert, cardiogenic shock	625	3.31	CT	-
22	Low-flow alert, cardiogenic shock	-	1.16	CT	-
23	Dyspnea	266	2.51	CT	-
24	Dyspnea	490	1.66	CT	-
25	None	228	3.74	CT	-
26	Low-flow alert	459	3.94	CT	-
27	Dyspnea	243	2.13	CT	-
28	Low-flow alert, dyspnea	292	2.56	CT	-
29	Low-flow alert, dyspnea, cardiac decompensation	423	2.92	CT	Severe
30	Dyspnea	235.8	2.56	CT	Mild
31	Low-flow alert	523.8	2.38	CT	Moderate
32	Low-flow alert	-	3.2	CT	Mild
33	Dyspnea	196	1.93	CT	Moderate
34	Low-flow alert	166.8	2.75	CT	Moderate
35	Dyspnea, low-flow alert	225	2.5	CT	Severe
36	Dyspnea	431	2.4	CT	Moderate
37	Dyspnea	335	1.1	CT	-
38	Dyspnea	392	1.6	PA	-
39	Low-flow alert	227.54	2.39	Echo	-

40	Low-flow alert	3.5	2.8		-
41	Dyspnea, right heart failure	-	2.1	CT	Moderate
42	Dyspnea, right heart failure	-	2	CT	Moderate
43	Dyspnea	360	2.9	CT	-
44	Low-flow alert	270	2.1	CT	-
45	Abdominal pain, nausea, vomiting (related to acute cholecystitis)	163	2.3	CT	Moderate
46	Low-flow alert	304	1.98	IVUS	Severe
47	Low-flow alert	292	2.7	CT	Moderate
48	Dyspnea	292	-	CT	-
49	Low-flow alert	-	-	CT	Severe
50	Low-flow alert	-	-	CT	Mild
51	Low-flow alert	-	-	CT	Mild
52	Low-flow alert	-	-	CT	Mild
53	Low-flow alert	-	-	CT	Mild
54	Low-flow alert	-	-	CT	Mild
55	Low-flow alert	-	-	CT	Mild
56	Low-flow alert	-	-	CT	Mild
57	Low-flow alert	-	-	CT	Mild
58	Low-flow alert	-	-	CT	Mild
59	Low-flow alert	-	-	CT	Mild
60	Low-flow alert	-	-	CT	Mild
61	Low-flow alert	-	-	CT	Mild
62	Low-flow alert	-	-	CT	Mild
63	-	-	-	CT	Mild
64	Low-flow alert, pulmonary congestion, deteriorating renal function	290	-	CT	-
65	Low-flow alert	596	-	CT	Moderate
66	Epistaxis	632	1.9	PA	Severe
67	Dyspnea, low-flow alert	534	1.4	CT	-
68	Low-flow alert, dyspnea, increasing diuretic requirements	481	-	CT	-
69	Dyspnea	229	3.5	PA	-
70	Hydropic decompensation	210	2.9	CT	Moderate
71	Dyspnea, hypoperfusion, low pump flow	-	-	CT	Severe
72	Fever, tachypnea, dyspnea, hypoxia	322.5	2.4	CT	-
73	Incidental finding/asymptomatic	-	-	CT	Moderate
74	Incidental finding/asymptomatic	-	-	CT	Mild
75	Incidental finding/asymptomatic	-	-	CT	Moderate

76	-	-	-	-	-
77	-	-	-	-	-
78	-	-	-	-	-
79	-	-	-	-	-
80	-	348	2.4	CT	Mild
81	Low-flow alert	200	2.4	CT	-
82	Low-flow alert	316	3.1	CT	Mild
83	Dyspnea, low-flow alert	199	3.4	CT	Severe
84	Dyspnea, low-flow alert	347	3	CT	Severe
85	Low-flow alert	291	2.1	CT	-
86	Low-flow alert	312	3.2	CT	-
87	Low-flow alert	312	3.2	CT	-
88	Low-flow alert	312	3.2	CT	-
89	Low-flow alert, syncope	-	-	CT	-
90	Low-flow alert, progressive dyspnoea, acute decompensated heart failure	-	-	CT	Severe
91	Cardiac arrest	-	-	CT	-
92	Cardiogenic shock, low-flow alert	-	-	CT	-
93	Low-flow alert	303	2.7	CT	Severe
94	Low-flow alert, dyspnea, hydropic decompensation	330	2.28	CT	Mild
95	Pulmonary oedema, low cardiac output	376	2.02	CT	Moderate
96	Dyspnea	290	2.15	CT	Moderate
97	Pulmonary congestion, peripheral oedema, dyspepsia	422	1.71	CT	Moderate
98	Dyspnea, ascites, heart failure	157	1.71	CT	Moderate
99	Pulmonary edema, hypotension	-	-	IVUS	Moderate
100	Dizziness, dyspnea, frequent low-flow alert	-	-	CT	-
101	Low-flow alert, new-onset heart failure, driveline exit site infection	-	-	CT	Severe
102	Dyspnea, angina pectoris	-	-	Echo	-
103	Low-flow alert, exertional dyspnea, dizziness	-	-	CT	Moderate
104	Low-flow alert, dyspnea, angina pectoris	-	-	CT	Moderate
105	Low-flow alert, incidental finding - patient evaluated for chronic driveline infection	-	-	Echo	Severe

106	Low-flow alert, murmur	392	-	CT	Severe
107	Low-flow alert, syncope	392	-	CT	Severe
108	Dyspnea, fatigue, murmur	392	-	CT	Moderate
109	Murmur	392	-	CT	Severe
110	Low-flow alert	392	-	CT	Severe
111	Low-flow alert, murmur	392	-	CT	Severe
112	Low-flow alert, dyspnea, dizziness, murmur	392	-	CT	Mild
113	Dyspnea, murmur	392	-	CT	Severe

Table S5: Clinical symptoms, diagnostic assessment and classification of eOGO, and laboratory parameters in individual patients. CT, computed tomography; eOGO, external compression of the outflow graft causing obstruction; Echo, echocardiography; PA, percutaneous angiography; IVUS, intravascular ultrasound.

Case	Therapy	Follow-up time (days)	Deceased
1	Surgery	1011	No
2	Stenting	744	No
3	Stenting	494	No
4	Stenting	85	Yes
5	Surgery	2	Yes
6	Surgery	200	No
7	Surgery	229	Yes
8	Stenting	127	No
9	Stenting	66	No
10	Surgery	18	No
11	Stenting	42	Yes
12	Transplantation	14	No
13	Wait	838	No
14	Wait	64	No
15	Surgery	573	No
16	Surgery	629	Yes
17	Surgery	551	Yes
18	Surgery	2	Yes
19	Surgery	677	No
20	Surgery	1069	No
21	Surgery	14	Yes
22	Surgery	755	Yes
23	Surgery	568	No
24	Transplantation	40	No
25	Transplantation	29	No
26	Transplantation	847	No
27	Surgery	175	No
28	Surgery	26	No
29	Surgery	747	No
30	-	219	Yes
31	Transplantation	135	No
32	Transplantation	30	No
33	Transplantation	250	No
34	Transplantation	3	No
35	Surgery	13	No
36	Stenting	317	No
37	Surgery	592	No
38	Stenting	173	No
39	-	0	Yes
40	Surgery	488	No
41	-	48	No
42	Transplantation	6	No

43	Surgery	320	No
44	Surgery	358	No
45	Wait	145	No
46	Stenting	131	No
47	Transplantation	923	No
48	Surgery	433.5	No
49	-	-	Yes
50	Surgery	365	No
51	-	365	No
52	-	365	No
53	-	365	No
54	-	365	No
55	-	365	No
56	-	365	No
57	-	365	No
58	-	365	No
59	-	365	No
60	-	365	No
61	-	365	No
62	-	365	No
63	-	365	No
64	Transplantation	305	No
65	Transplantation	387	No
66	Stenting	733	No
67	Stenting	183	No
68	Stenting	914	No
69	Stenting	18	Yes
70	Wait	3	No
71	Surgery	365	No
72	Intravenous anticoagulation	2	Yes
73	Wait	2206	No
74	Wait	2052	No
75	Wait	1767	No
76	-	-	No
77	-	-	No
78	-	-	No
79	-	-	-
80	Surgery	-	-
81	Transplantation	-	No
82	Surgery	2	Yes
83	Surgery	978	No
84	Surgery	282	No
85	Surgery	5	Yes

86	Stenting	4	No
87	Stenting	4	No
88	Stenting	4	No
89	Surgery	7	No
90	Stenting	4	Yes
91	Stenting	1	Yes
92	Stenting	1	Yes
93	Stenting	609	No
94	Stenting	637	No
95	Stenting	536	No
96	Stenting	457	No
97	Wait	487	No
98	Stenting	24	Yes
99	Surgery	-	No
100	Stenting	-	No
101	Surgery	55	No
102	Stenting	31	No
103	Surgery	275	No
104	Surgery	-	-
105	Stenting	-	No
106	Surgery	732	No
107	Transplantation	732	No
108	Surgery	732	No
109	Surgery	732	No
110	Stenting	732	No
111	Transplantation	732	No
112	Transplantation	732	No
113	Surgery	732	No

Table S6: eOGO treatment strategy, follow-up time in days, and outcome of treatment in individual patients included in this review. eOGO, external compression of the outflow graft causing obstruction.

**Diagnostic parameter**			**Patients with available data, *n* (%)**	
	Admission	Discharge	Admission	Discharge
Laboratory parameter, median [IQR]				
Serum lactate dehydrogenase (U/L)	303.5 [227.7, 392.0]	254.0 [223.5, 305.8]	74 (65.5)	44 (38.9)
INR	2.4 [2.0, 2.8]	2.1 [1.8, 2.6]	67 (59.3)	47 (41.6)
LVAD parameter, median [IQR]				
Flow (L/min)	3.4 [2.5, 4.5]	4.5 [4.2, 5.0]	63 (55.8)	49 (43.4)
Motor power (W)	4.0 [3.7, 4.4]	4.2 [3.9, 4.5]	51 (45.1)	43 (38.1)

Table S7: Laboratory and LVAD parameters of eOGO patients. INR, international normalized ratio; LVAD, left ventricular assist device; eOGO, external compression of the outflow graft causing obstruction.

**LVAD parameter, median [IQR]**	**Stenting**	**Surgery**
Flow at admission (L/min)	3.30 [2.28, 4.10]	3.00 [2.40, 4.00]
Flow at discharge (L/min)	4.50 [4.30, 4.80]	4.80 [4.30, 5.15]
Motor power at admission (W)	4.00 [3.60, 4.30]	3.85 [3.68, 4.40]
Motor power at discharge (W)	4.15 [3.77, 4.30]	4.35 [4.05, 4.77]

Table S8: LVAD parameters of eOGO patients divided into therapy subgroups stenting and surgery. LVAD, left ventricular assist device; eOGO, external compression of the outflow graft causing obstruction.
